# A Case of Fracture, Attended with Symptoms of Unusual Violence, Relieved by an Extensive Longitudinal Incision through the Fascia of the Limb

**Published:** 1814-03

**Authors:** John Mackesy

**Affiliations:** Surgeon, 62d Regt. Palermo


*For the Medical and Physical Journal.*

*A Case of Fracture, attended with Symptoms of unusual Violence, relieved by an extensive longitudinal Incision through the Fascia of the Limb; by* Mr. Mackesy, of Palermo.

THE severity of symptoms, and formidable appearance which marked the following case of simple fracture, together with the mode of treatment found effectual in their relief, relief, may entitle the particulars relative to it to more attention than such occurrences usually demand.

John Creighton, private in the 1st battalion 62d regiment, aged 18, of no remarkable temperament, was admitted into the regimental hospital on the morning of the 19th of April, 1813, with simple fracture of the tibia and fibula in the small of the leg, near the ancle; but as tumefaction had already set in, the exact place of fracture could not be accurately ascertained. No very remarkable pain or symptom was present. The knee being moderately bent, the limb was laid on its outside in an easy relaxed posture, the many-tailed bandage was used, and the limb dressed in the usual manner; a weak saturnine solution was ordered to be occasionally applied; the tapes of the splints were loosely tied.

April 20th.—Violent pain of the limb came on in the course of the night, the swelling and tension had considerably increased; inflammatory fever, with its usual concomitants, a hot dry skin, florid countenance, quick hard full pulse had commenced. Forty ounces of blood were immediately discharged, and ℥ifs Sulphat. Sodæ given; the tapes were thrown open, the upper splint removed, and the tails of the bandage undone. The saturnine solution continued.

21st.—The pain of the limb was increased; fever very considerable; the neighbourhood of the fracture exhibited a slight livid appearance; and several vesicles had already formed round the injured part.

℞. Fiat Venæsectio ad ℥xxxxvj.

Habeat Vespere Tincturæ Opii gtt. xxx.

22d.—The pain and fever were more moderate; he slept a little during the night; the vesicles had become larger and more numerous.

℞. ℥j Sulphat. Magnesiæ statim.

23d.—Every distressing symptom had now returned with redoubled violence; the pain and tension of the limb were so excruciating as to produce the greatest torture; the livid color was evident all round the injury; each vesicle was become more enlarged, with a dark base; and they now extended over the instep to the points of the toes; every appearance indicated the near approach of gangrene, and the occurrence of tetanus was apprehended from the violence of the patient's sufferings.

Such were the threatening symptoms on the morning of the 23d, when, with a common scalpel, a longitudinal incision was made, about nine inches long, on the inside of the limb, commencing at the swell of the gastrocnemii muscles, passing directly over the malleolus internus, and terminating in the sole of the foot. It was of sufficient depth in some part of its course to divide the fascia. There was immediate retraction of the tense integuments, and two small branches of an artery sprung, which were allowed to stop of themselves. The relief in consequence was immediate: the violent pain and burning heat instantly subsided, and the poor creature was now removed from a state of the utmost torture to that of comparative felicity. Sixteen ounces of blood were taken from the arm, a well-made emollient poultice was applied, and the patient left to repose.

24th.—Morning visit. Had a good night's rest; free from pain, and the febrile symptoms by no means so considerable.

The sequel of this case is too unimportant to merit minute detail; it is enough to observe in general terms, that from this date there was no return of pain, or of any alarming symptom. The fever had quietly subsided; the vesicles broke, and discharged their ichor; the hard cuticle peeled off; the bones united; and the scar healed in the usual manner. The patient was discharged to duty on the 30th of June, without the limb suffering under any remaining deformity.

When the tumefaction of the injured part was reduced, the form and direction of the fracture could be traced, and were peculiar. In the fibula it was transversely, about two and a half inches above the joint.

The seat of fracture in the tibia could be distinctly felt in the shape of a groove on that face of the bone between the malleolus internus and the sharp angle which forms the shin: it descended into the joint, and proceeded upwards towards the knee, along the length of the tibia about seven inches, sloping gradually outwards towards its termination, giving to the upper part of the fracture a slender scolloped point. The violent attendant symptoms may be justly attributed to this peculiarity of the fracture—its extending into the joint, and continuation along the length of the bone.

In the present improved state of the practice of surgery, it would be presumptuous in me to claim any merit for an idea which has occasionally directed a similar mode of treatment even in the rudest periods of the profession. My object in marking the case is to rescue from oblivion an important fact; and to point out the utility of a bold extensive incision, even in simple fracture, where, from the violence of inflammation or excess of irritation, suppuration or gangrene are threatened to ensue. The manner of its acting in liberating the suffering part, is too obvious to require observation.

Nor does this mode of proceeding militate against the ideas of the celebrated Pott. His disapprobation is directed against a superficial incision, or trifling scratching of the surface, surface, which to a limb in high action must inevitably add to every distressing symptom.

Before I conclude what may be deemed an important subject, I would hazard the opinion that in such cases as the present, the much talked of and much blamed fascia is not so much in fault. I should rather attribute the binding and stricture of the injured part, in a great degree, to the rigid unyielding state of the external integuments, now rendered tense and turgid with blood and serum, from increased vascular action. This, however, must greatly depend on the structure of the part where the injury may be situated.

*Palermo,* JOHN MACKESY,

